# Malaria and protective behaviours: is there a malaria trap?

**DOI:** 10.1186/1475-2875-12-200

**Published:** 2013-06-13

**Authors:** Jean-Claude Berthélemy, Josselin Thuilliez, Ogobara Doumbo, Jean Gaudart

**Affiliations:** 1 CES-CNRS, Université Paris 1, Panthéon-Sorbonne, Centre d'économie de la Sorbonne, Maison des Sciences Economiques, 106-112 Boulevard de l'Hôpital, 75013, Paris, France; 2 FERDI, Fondation pour les études et recherches sur le développement international, 63 boulevard François-Mitterrand, 63000, Clermont Ferrand, France; 3 Malaria Research and Training Center (MRTC),Université du Mali, Faculté de Médecine, de Pharmacie et d’Odonto-Stomatologie, Département d’épidémiologie des affections parasitaires (DEAP). UMI3189, BP1805, Bamako, Mali; 4 Aix-Marseille University, UMR912 SESSTIM (INSERM IRD AMU), 13005, Marseille, France

**Keywords:** Protective behaviours, Poverty, Economic epidemiology, Malaria, ITN

## Abstract

**Background:**

In spite of massive efforts to generalize efficient prevention, such as insecticide-treated mosquito nets (ITN) or long-lasting insecticidal nets (LLINs), malaria remains prevalent in many countries and ITN/LLINs are still only used to a limited extent.

**Methods:**

This study proposes a new model for malaria economic analysis by combining economic epidemiology tools with the literature on poverty traps. A theoretical model of rational protective behaviour in response to malaria is designed, which includes endogenous externalities and disease characteristics. Survey data available for Uganda provide empirical support to the theory of prevalence-elastic protection behaviours, once endogeneity issues related to epidemiology and poverty are solved.

**Results:**

Two important conclusions emerge from the model. First, agents increase their protective behaviour when malaria is more prevalent in a society. This is consistent with the literature on "prevalence-elastic behaviour". Second, a ‘malaria trap’ defined as the result of malaria reinforcing poverty while poverty reduces the ability to deal with malaria can theoretically exist and the conditions of existence of the malaria trap are identified.

**Conclusions:**

These results suggest the possible existence of malaria traps, which provides policy implications. Notably, providing ITN/LLINs at subsidized prices is not sufficient. To be efficient an ITN/LLINs dissemination campaigns should include incentive of the very poor for using ITN/LLINs.

## Background

Historically, in absence of protection tools, malaria persisted in large regions of the world [1,2]. Economic development is also linked to malaria elimination and the bidirectional relationship between malaria and development has been extensively discussed in the economic literature [3-6]. More recently it has been shown that insecticide-treated mosquito nets and long-lasting insecticidal nets (ITN/LLINs) are efficient preventive tools [7-11], and this has triggered ambitious campaigns of ITN/LLINs dissemination, with the expectation that such campaign would help eliminate/eradicate malaria [12,13]. However, in spite of their efficiency, ITN/LLINs are only partially used for malaria prevention by the populations, and this behaviour could hinder malaria elimination/eradication [13]. A malaria trap defined as the result of malaria reducing economic output (reinforcing poverty), while poverty reduces the ability to deal with malaria, could be one possible explanation of this paradox as, even in countries with high risk populations, the use of ITN remains low [13,14].

In recent economic literature [15], it has been argued that there could exist a poverty trap associated with a dynamic interaction between a disease prevalence and poverty: disease prevalence increases poverty, while poverty increases the susceptibility to infectious diseases. However, this approach has been essentially based on empirical estimates of macroeconomic relations between income GDP per capita (Gross Domestic Product) and infectious disease burden (DALYs, Disability Adjusted Life Years). This kind of result has been used to advocate disease protection campaigns, e.g. distribution of ITN/LLINs at subsidized prices [16]. Following this line of arguments, the existence of a malaria trap, as previously defined, is plausible. Analysing the negative impact of the disease on productivity [17-19] provides some empirical evidence of the influence of malaria on poverty, although the magnitude of this influence is debated. The link between malaria and poverty may be also more indirect. Indeed, it has been shown empirically that malaria significantly reduces children cognitive capacity [20,21]; hence, malaria can prevent extreme poverty eradication, insofar as education is one of the basic ingredients of poverty alleviation policies. Notably, standard pro-poor policies, such as the development of publicly-subsidized primary education, may fail in regions where the prevalence of malaria is high [22]. Thus the assumption of a malaria trap in regions characterized by extreme poverty with low educational attainment and high malaria incidence should be seriously considered. In the presence of a malaria trap, standard pro-poor policies that are usually advocated, such as the subsidy of protection devices on the one hand, and the subsidy of education on the other hand, may fail. The aim of this work was to develop a model combining an analysis of human protective behaviours with a classical representation of the epidemiological malaria transmission, and to test the conclusions of this model.

## Methods

In order to evaluate the poverty related-malaria trap, the basic epidemiological model of malaria transmission, in absence of protection, was reminded. Second, an economic model was developed on the basis of the epidemiological one, studying model’s behaviour at steady-state. Third, predictions of the model was tested based on Demographic and Health Survey data from Uganda [23].

### Epidemiological model in absence of protection

A standard epidemiological model of malaria was built, with transmission of malaria between a population of humans and a population of mosquitoes [24]. In order to simplify the framework, the usual assumptions have been made, i.e. constant population sizes (human and mosquito) over time, uniform contacts between human and mosquitoes, ignorance of superinfection and immunity. Within the life-time period of humans, malaria prevalence among humans and mosquitoes reaches a steady state. This leads, in absence of protection, to equations based on the McDonald and Ross malaria transmission model [24].

The time variation of malaria prevalence among humans can be defined in a simplified way as:

(1) $$\dot{X}=\text{mabZ}\left(1-X\right)-\mathrm{rX}$$

where *m* is the vector density (ratio of mosquitoes per human), *a* is the number of bits per unit of time and per mosquito, *b* is the proportion of infected bites that produce infection among humans, *Z* is the proportion of infectious mosquitoes, and *r* is the clearance rate of malaria in humans.

Similarly, the time variation of the proportion of infectious mosquitoes, can be written as:

(2) $$\dot{Z}=\text{acX}\left(e^{-\mathrm{gn}}-Z\right)-\mathrm{gZ}$$

where *c* is the proportion of bites on infectious humans that produce infection among mosquitoes, *g* is the death rate of mosquitoes, and *n* is the length of sporogonic cycle.

Assuming that the time period of life is long enough, malaria prevalence reaches a steady state equilibrium defined by [24]:

(3) $$Q\left(X\right)=\frac{\text{mab}\frac{\text{acX}e^{-\mathrm{gn}}}{g+\text{acX}}}{r+\text{mab}\frac{\text{acX}e^{-\mathrm{gn}}}{g+\text{acX}}}=\frac{\text{bEIR}}{r+\text{bEIR}}$$

where EIR is the entomological inoculation rate classically defined such as:

$$\text{EIR}=\text{maZ}=\frac{ma^{2}\mathrm{cX}e^{-\mathrm{gn}}}{g\;+\;\text{acX}}$$

In what follows (after protection through ITN/LLIN), the parameter *m* will become itself a variable. The function *Q(X, m)* is concave, and characterized by the following properties:

$$\left\{\begin{array}{c} Q\;\left(0,m\right)=0\\ Q\;\left(1,m\right)<1 \end{array}\right)$$

And its slope at origin, is equal to

(4) $$\frac{\partial Q\;\left(0,m\right)}{\partial X}=\frac{ma^{2}\mathrm{bc}e^{-\mathrm{gn}}}{\mathrm{rg}}=R_{0}$$

This number, *R*_0_, is classically called, in the McDonald and Ross tradition, the “basic reproduction number” [25,26]. As demonstrated elsewhere, if *R*_0_ is below or equal to 1, then *Q(X,m)* converges towards the trivial disease free stable steady state. This case is not considered in what follows, as it does not coincide with the persistence of malaria in large regions of the developing world. Conversely, if *R*_0_ is higher than 1, then *Q(X,m)* converges towards a stable steady state characterized by a strictly positive prevalence of malaria. In what follows, *Q(X,m)* will be considered as the functional relationship between the prevalence and contagious persons, at steady state, also depending on the vector density *m*. This vector density will depend on protection behavior against mosquitoes.

### Economic epidemiological model with protection

When the basic reproduction number *R*_0_ is higher than 1, using protection tools could nevertheless reduce malaria transmission [24], and then, the trivial disease free stable steady state could be reached. This is the rationale of ITN/LLINs dissemination policies. In order to assess this possibility, a model of protection behavior has been added to the previous epidemiological model. This behavioral model, as described below, is based on economic mechanisms. Peoples adopt a certain behavior: use of an insecticide-treated net (*h* = 1) or exposure to malaria risk (*h* = 0). It is supposed that the only means by which a person can prevent himself from parasitic infection is to sleep under an ITN/LLIN (even if a person can be infected during the first part of the night). As a first assumption, the use of an ITN/LLIN was supposed to provide complete protection from malaria infection. This assumption has been relaxed in Additional file 1 without affecting the main findings of the model. At any time, depending on the use of ITN before, the health status of the individual, *σ*(*h*) can take one of two values: susceptible, *σ*(*h*) = *S*, or infected, *σ*(*h*) = *I*. The probability of being infected at any time, conditionally to the absence of protection before, can then be written as:

(5) $$\pi_{I}=P\left(\sigma \left(h\right)=I/h=0\right)$$

If *H* is the proportion of population using ITN/LLIN, among the (*1-X)* uninfected persons, the proportion of infected persons can be simply written as:

(6) $$X=\left(1-H\right)\pi_{I}$$

Furthermore the density of mosquitoes in contact to humans, is affected by the presence of ITN/LLIN used by a proportion *H* of the population. First, as the contact between mosquito and human is more difficult, the denominator of the mosquito density decreases, being now the proportion 1 – *H* of non-protected population. Second, as ITN/LLINs do not only protect humans, from anopheles bites, but also kill mosquitoes (knock down effect), the numerator (the number of mosquitoes) decreases with *H*. Hence *m* can be written as follows:

(7) $$m\left(H\right)=\frac{m\left(0\right)}{1-H}\left(1-\gamma \left(H\right)\right)$$

Where *γ*(*H*) is the proportion of mosquitoes killed by the use of ITN/LLINs, an increasing function of *H*. Note that, the EIR is then a decreasing function of *H*. It follows that, at the steady state:

(8) $$\pi_{I}=Q\left(X,m\left(H\right)\right)$$

In order to complete the model, the determinants of *H* were specified in a next step. At the microeconomic level, the choice of protection is determined by maximizing the expected utility of each individual. The decision *h* of protection (*h* = 1 for protection, *h* = 0 for non-protection) affects individuals’ utility through two channels: (i) an expected positive impact on his/her health status in case of protection and (ii) a private cost, called *κ*. This cost can be interpreted as the shortfall of paying for protection. This broad definition includes the opportunity cost of protection and depends on the marginal utility of personal income. In other words, the private cost of the ITN/LLIN, includes direct, indirect and opportunity costs, such as paying the market price of ITN/ILLNs (direct costs), transportation costs to get them or costs related to social ostracism (indirect costs). Opportunity costs are the costs of using them for protection rather than using them for an economically productive activity, or the cost equal to what an individual must give up in order to use an ITN/LLIN, which he/she would otherwise never use. Hence protection decision is described through the following maximization program:

(9) $${\text{max}}_{h}E\left[u\left(\sigma \left(h\right)\right)\right]-\mathrm{\kappa W}\left(\omega \right)h$$

Where *u* (*S*) or *u* (*I*) are the utility levels attached to the health status (susceptible, *σ*(*h*) = *S*, or infected, *σ*(*h*) = *I*, thus depending on *h*, the use of a protection), with 0 < u(I) < u(S); ω is the individual income; *W* (*ω*) is the marginal utility of the income, supposed as usual to decrease with income [27], and κ is the private cost of the ITN/LLIN.

The expected utility (the expected positive impact of using ITN/LLIN on the health status) can be estimated using the following probabilities of being susceptible or infected, conditionally to the use of protection:

(10) $$\begin{array}{c} \mathrm{st}\\ \begin{array}{l} P\left(\sigma \left(h\right)=S/h\;=\;1\right)=1\\ P\left(\sigma \left(h\right)=S/h\;=\;0\right)=1-\pi_{I}\\ P\left(\sigma \left(h\right)=I/h=0\right)=\pi_{I\;} \end{array} \end{array}$$

In addition, it is assumed that there exists a minimum subsistence level such as in the case a Stone-Geary utility function [28-30]. This implies that the marginal utility of income *W* (ω) goes to infinity for all individuals at (or below) the minimum subsistence level, which is classically called the extreme poverty line Ω (i.e. the minimum level of income deemed adequate in a given country for an individual or a household). In other words, the extreme poverty line is an income level below which nobody can afford an ITN/LLIN, i.e. h = 0.

As in standard economic epidemiological models, the individual will use protective tools when *W* (ω) is lower than the expected utility loss associated with the risk of infection that occurs in the absence of protection:

(11) $$E\left[u\left(\sigma \left(1\right)\right)-u\left(\sigma \left(0\right)\right)\right]\geq \;\mathrm{\kappa W}\left(\omega \right)$$

According to Equation (9) and the three probabilities of Equation (10) it follows that:

(12) $$h=1\;\text{if}\;\text{and}\;\text{only}\;\text{if}\;u\left(S\right)-\left(1-\pi_{I}\right)u\left(S\right)-\pi_{I}u\left(I\right)\geq \mathrm{\kappa W}\left(\omega \right)$$

A person will use ITN/LLIN if the utility of being non-infected is greater than the utility of paying for a protective tool, according to the income and the probability of being infected without using any protection. Hence, protection occurs if and only if:

(13) $$\pi_{I}\geq \frac{\mathrm{\kappa W}\left(\omega \right)}{u\left(S\right)-u\left(I\right)}$$

This Equation shows that there is a threshold probability of infection above which a person engages in protection. The key point in this approach is that the threshold probability of infection depends on the marginal income utility loss associated with using the ITN/LLIN, *κW*(*ω*)*,* with respect to the net value attached to susceptible health status, u(S)–u(I). This threshold depends on the individual income *ω*. The threshold function, linking *π*_*I*_ to *ω*, termed *C*(*ω*)*,* is monotonic and *C* ' (*ω*) < 0, as the function *W*() is monotonic and *W* ' (*ω*) < 0. In addition, the function *C*() is increasing with κ. Consequently:

(14) $$\left\{\begin{array}{l} h=1\;\text{if}\;\omega \geq C^{-1}\left(\pi_{I}\right)\\ h=0\;\text{else} \end{array}\right)$$

and the income threshold conditioning protection, *C*^− 1^(*π*_*I*_), decreases with κ. Knowing individual protection behaviors, the aggregated level of protection *H* (the percentage of protected persons) can be computed by integration as follows:

(15) $$H=\int_{C^{-1}\left(\pi_{I}\right)}^{+\;\infty }f\left(\omega \right)\mathrm{d\omega}=1-F\left(C^{-1}\left(\pi_{I}\right)\right)$$

Where *f* is the probability density function of *ω* (and *F* the associated cumulative density function), describing the income distribution of the population. Equations (6), (8) and (15) fully describe the dynamics of *H* and *π*_*I*_ as a function of *X*.

### Prevalence-elastic behaviour at the steady-state vicinity

Nearby the steady-state, the dynamics corresponds to a standard prevalence-elastic behaviour of protection (positive malaria prevalence elasticity), where *H* is an increasing function of *X*, because it is increasing with *π*_*I*_ (Equations (8) and (15)). Note that as a consequence, nearby the steady-state, *X* is not necessarily monotonic in *π*_*I*_: protection behaviors and epidemiological dynamics go in opposite directions. Indeed, combining Equations (6) and (15) it follows that:

(16) $$X=F\left(C^{-1}\left(\pi_{I}\right)\right)\pi_{I}$$

As a result, this is consistent with standard results in economic epidemiology [31]. Thus, Equations (15) and (16) provide us with some economic determinants of protection at individual and aggregated levels, that could be possibly tested (as studied in next section). For a given probability of infection in absence of protection, protection decreases with the unit costs of ITN/LLIN, κ (through the function *C*^− 1^). It also decreases with poverty, as the poorer the individuals, the higher their marginal utility of income.

### Long term properties: conditions of persistence of a malaria trap

The main question to be solved, concerning the long-term properties of this model at the steady-state, is whether a malaria trap can persist in the long run, in spite of the availability of ITN/LLINs as protection tools since the higher the unit cost κ of ITN/LLINs, the lower the protection. This is why ITN/LLINs programs are usually based on subsidized ITN/LLINs prices. Let us then consider the best case of almost full subsidization, when κ*→*0 (i.e. the extreme case being free distribution). Conditions under which, for any positive unit cost κ, the malaria trap persists are given below:

### Proposition

For any κ >0, when κ*→*0 the long term equilibrium corresponds to a malaria trap, if and only if:

(17) $$R_{0}>\frac{1}{F\left(\Omega \right)\left(1-m\left(F\left(\Omega \right)\right)\right)\;}$$

where F(Ω) is the proportion of persons under the extreme poverty line in a population, also called the extreme poverty incidence. Note that *m* depends on *H*, the proportion of protected persons (Equation (7)), which depends itself on income (Equation (15)), and, thus, on the extreme poverty incidence.

### Proof

For *κ* → 0, individuals use protection if and only if they are above the extreme poverty line. Given Eq. (6), (8) and (15), it follows that:

*H* → 1 − *F*(*Ω*), i.e. only persons over the extreme poverty line will be protected then, *X* → *F*(*Ω*)*π*_*I*_ i.e. only persons under the extreme poverty line will be infected at rate *π*_*I*_

and

(18) $$\pi_{I}\to Q\left(X,m\left(1-F\left(\Omega \right)\right)\right)$$

Thus, when κ*→*0, the long term equilibrium can be mathematically analyzed along the same line as in the pure epidemiological model at the steady-state, after substitution of function *Q* by:

(19) $$F\left(\Omega \right)Q\left(X,m\left(1-F\left(\Omega \right)\right)\right)$$

This implies that the malaria trap persists if and only if the slope of this function at origin (X = 0) is higher than 1, i.e.:

(20) $$R_{0}=F\left(\Omega \right)\frac{m\left(1-F\left(\Omega \right)\right)a^{2}\mathrm{bc}}{\mathrm{rg}}>1\;\text{QED}$$

Given *κ* → 0 and *H* → 1 − *F*(*Ω*), as the vector density *m* is a decreasing function of *H*, the higher the incidence of extreme poverty *F*(*Ω*), the higher the risk of persistence of a malaria trap. Hence, a malaria trap will persist for high enough values of the basic reproduction number *R*_0_ and of the extreme poverty incidence *F*(*Ω*), even when ITN/LLINs are highly subsidized. In the extreme case where all the population is at or below the extreme poverty line, the condition above corresponds exactly to the basic reproduction number, and hence this policy is certainly ineffective (*R*_0_ > 1).

One could argue that if ITN/LLINs were provided at no cost to individuals (κ = 0), then all individuals including the extreme poor, would use them. Distribution of ITN/LLINs for free would then possibly be a much more efficient policy to reduce malaria, compared to selling ITN/LLINs at a subsidized price. This is in line with randomized experiments that found that free distribution dramatically increases use of ITN/LLINs (as well as other important products for the poor), compared to charging even very small user fees [32].

However, the assumption *κ* = 0 is merely theoretical, even though it can be possibly obtained in controlled experiments, as in practice *κ* is not merely the price of ITN/LLINs: it involves also all opportunity costs attached to using them for other productive activities. Selling, exchanging, discarding or re-using the material from ITN/LLINs is not uncommon. For instance, misuse of ITN/LLINs for profit (drying fish and fishing) has been observed by Lake Victoria [33] and in Zambia [34]. In some cases, nets have even been turned into wedding dresses and water filters.

### Predictability of the model

**Figure 1:**
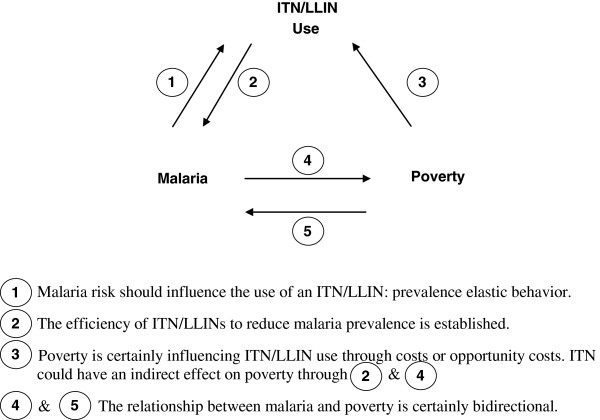
The relationship between ITN/LLINs use, malaria and poverty.

The previous model describes protection behaviours and the existence of theoretical conditions under which a malaria trap persists. As stated above, protection should (a) increase with prevalence of malaria (i.e. positive malaria prevalence elasticity), (b) decrease with an increase of economic cost of protection and (c) decrease with an increase of the incidence of extreme poverty. Prediction (a) is testable with existing individual data as examined below. It corresponds to relation (1) of Figure 1. Prediction (b) is hardly testable for lack of data on cost of protection at individual level. However, prediction (c) is testable, and provides a way to study economic determinants of protection. Finally, the model predicts that, in a country with high extreme poverty incidence, the policy of dissemination of subsidized ITN/LLINs, sold at a small but strictly positive unit price, can be ineffective, insofar as the corrected basic reproduction number is increasing with extreme poverty incidence. As classically done, three regression models (ordinary least square estimators, OLS) have been used to estimate explanatory variables of (i) poverty, (ii) malaria prevalence, (iii) use of protection. Each of these 3 models includes, as predictor, the two remaining variables, plus cofactors to be adjusted on. But, none of these models takes into account endogeneity, which leads to estimation bias. Endogeneity is used in economics to describe the presence of an endogenous explanatory variable in a multiple regression model, i.e. a variable that is correlated with the error term, either because of an omitted variable, measurement error or reverse causality [35]. Indeed, classical regression models explaining malaria prevalence and protection use are certainly biased due to endogeneity. As illustrated in Figure 1 reverse causality is actually a highly plausible bias. In order to solve this problem, instrumental variables techniques have been used to deal with these potential biases [35-38]. Consequently, given the strong possibility of endogeneity of poverty incidence, malaria prevalence and protection, and given that the error terms are not necessarily independent of each other, the 3SLS estimates are useful, providing estimates that are free of endogeneity bias. The complete system of structural equations was estimated with a heteroskedastic-efficient 3SLS two step generalized method of moments (3SLS GMM) described by Wooldridge [35]. The system is illustrated in a simplified way with Equation (21) below and Figure 1:

(21) $$\left\{\begin{array}{l} F\left(\Omega \right)=\alpha_{1}+\beta_{1a}X+\beta_{1b}\;\text{Poverty}\;\text{Incidence}\;\text{IVs}+\beta_{1c}\;\text{Regions}+\epsilon_{1}\\ X=\alpha_{2}+\beta_{2a}F\left(\Omega \right)+\beta_{2b}H+\beta_{2c}\;\text{Malaria}\;\text{IVs}+\beta_{2d}\;\text{Regions}+\epsilon_{2}\\ H=\alpha_{3}+\beta_{3a}X+\beta_{3b}F\left(\Omega \right)+\beta_{3}{}_{c}\;\text{Protection}\;\text{IVs}+\beta_{3d}\;\text{Regions}+\epsilon_{3\;} \end{array}\right)$$

Where the *βs* are the coefficients associated with the corresponding factors (or vectors of factors) included in each equation, *IV*s stand for specific Instrumental Variables enabling to identify a causal effect (described below), *Regions* are a set of dummy variables in order to control for confounders linked to malaria regional control programs, and *ϵs* are the standard disturbance terms. Other variables remain unchanged with respect to previous notations.

Instrumental variables for endogenous variables are distance to nearest market and remote location (for poverty: the poorest of the poor), altitude, longitude, latitude (for malaria prevalence), distance to nearest health center, % of houses sprayed against mosquitoes in last 12 months (for ITN use). Similar geographic instruments were already used for malaria prevalence [5,20] and the availability of health facilities is generally a good candidate as instrumental variable for endogenous health indicators [39]. Household distance to nearest market is supposed to independently affect economic activity and resources as in other examples. In a three-equation model, the order condition for identification requires that there is at least two endogenous or exogenous variables excluded from each equation, which is the case here [40]. The Hansen *J* test was used to test the over-identifying restrictions i.e. the validity of the instrumental variables [35]. A rejection of the null hypothesis implies that the instruments are not satisfying the orthogonality conditions required for their employment (i.e. they are uncorrelated with the error term of the estimated equation). The predictions of the model have been tested on DHS (Demographic and Health Surveys) household members data from Uganda (2009) aggregated at the community/village level. Note that DHS are generally not longitudinal surveys. Unfortunately, the selected clusters or individuals within a same country generally change from one survey to another. To date, MIS (Malaria Indicator Surveys), that provide at the same time malaria infection prevalence (through Paracheck tests) and ITNs use for children under five, are extremely recent and cover only five African countries [41]: Liberia (2008–2009) Angola (2006), Senegal (2008–2009), Uganda (2009) and Tanzania (2007–2008), hence Ugandan data were used [23]. The choice of the survey highly depends on the kind of information available in each survey. Potential reliable instrumental variables were not available in Angola, Liberia, Tanzania and Senegal. In Uganda, 28.67% of the population lives with less than the threshold of $1.25 a day (in PPP [42]). The extreme poverty line was, therefore, defined using this threshold and the wealth index provided by DHS. Poverty incidence was calculated/estimated relatively to this extreme poverty line. Malaria prevalence was defined as the percentage of people tested positive for malaria with RDTs amongst the population tested in the survey. An ever-treated net is (i) a factory-treated long-lasting insecticidal mosquito net that does not require any further treatment, OR (ii) a factory net, with or without an insecticide kit, which has subsequently been soaked with insecticide at any time, OR (iii) a homemade net which has subsequently been soaked with insecticide at any time. Note that ordinary least-square models (OLS) have been kept in order to highlight the endogeneity bias.

### Application results

Table 1 shows that reasons generally advanced in the literature for not using the nets, do not hold in the Uganda DHS report where these questions were asked. The main reason of not using nets was the heat (for 15% of the sample). Note that, ITN/LLIN are more used against nuisance of *Culex* or *Aedes* than *Anopheles*. OLS regression (Table 2), not taking into account endogeneity, showed no significant prevalence-elastic behaviour (i.e. relationship (1) of Figure 1): protection behaviour was not significantly related to malaria prevalence, nor poverty incidence (Table 2 column 1), adjusted on regions, but malaria prevalence was significantly related to poverty incidence (β=0.376, *p*<0.001) (Table 2 column 2 and 3). Furthermore, protection behaviour changed through region, as well as malaria prevalence and poverty incidence. Conversely, once the endogeneity problems were solved, a prevalence-elastic relationship between malaria and protection was found: protection behaviour was significantly related to malaria prevalence (β=0.438, *p*=0.012), and negatively related to poverty incidence (β=−0.323, *p*=0.096) (Table 2, column 4). Malaria prevalence remains significantly related to poverty incidence at a higher magnitude (β=0.543, *p*<0.001). Prevalence-elastic behaviour was defined in economic epidemiology as an increased protection behaviour in response to an increase in disease prevalence. Hence one of the questions tackled previously was how changing malaria affects protection? Regression (4) of Table 2 thus confirmed a significant causal positive effect of malaria on protection (ITN use), as far as the instrumentation strategy (use of instrumental variable for adjustments) is validated by usual tests. The Hansen *J* Test of over-identifying restrictions showed that the instruments were valid in 3SLS GMM regressions (Table 2).

**Table 1:** Subjective reasons for not using the nets in Uganda

**Reason for not using the nets:**	**Too hot**		**Don't like smell**		**No mosquitoes**		**Net too old**	
	**Yes**	**No**	**Yes**	**No**	**Yes**	**No**	**Yes**	**No**
	15.7%	84.3%	1.6%	98.4%	6.8%	98.31%	11.4%	88.6%

Percentage of households with at least one mosquito net that was not slept under the previous night, and among those, percentage reporting various reasons for not using a net for sleeping the previous night, by background characteristics [23].

**Table 2:** Prevention, malaria and poverty in Uganda: OLS and 3SLS GMM regressions results

	**(1)**	**(2)**	**(3)**	**(4)**	**(5)**	**(6)**
	**OLS**	**OLS**	**OLS**	**3SLS GMM**		
	**Dep Var is % using an ever treated net last night in the village**	**Dep var is malaria prevalence in the village**	**Dep var is poverty incidence in the village**	**Dep Var is % using an ever treated net last night in the village**	**Dep var is malaria prevalence in the village**	**Dep var is poverty incidence in the village**
Malaria	-	−0.044	-	-	−0.862***	-
Prevention		(0.130)			(0.308)	
Poverty	−0.046	0.376***	-	−0.323*	0.543***	-
Incidence	(0.078)	(0.096)		(0.194)	(0.132)	
Malaria	−0.022	-	0.242***	0.438**	-	0.302**
Prevalence	(0.063)		(0.067)	(0.175)		(0.124)
Intercept	0.208***	0.400***	−0.013	0.062	1.364***	−0.290***
	(0.044)	(0.058)	(0.047)	(0.078)	(0.174)	(0.064)
Observations	170	170	170	170	170	170
R-squared	0.379	0.550	0.668	-	-	-

The coefficients attached to each variable are presented (standard errors, adjusted for heteroscedasticity in parentheses). All regressions include regional dummies. ***denotes statistical significance at the 1% level, ** at the 5% level, * at the 10% level. The Hansen J Test of overidentifying restrictions shows that the instruments are well identified in 3SLS GMM regressions (Hansen's J chi2= 12.247; *p* value = 0.140). A rejection of the null hypothesis implies that the instruments are not satisfying the orthogonality conditions required for their employment (i.e. that they are uncorrelated with the error term of the estimated Equation).

## Discussion

**Figure 2:**
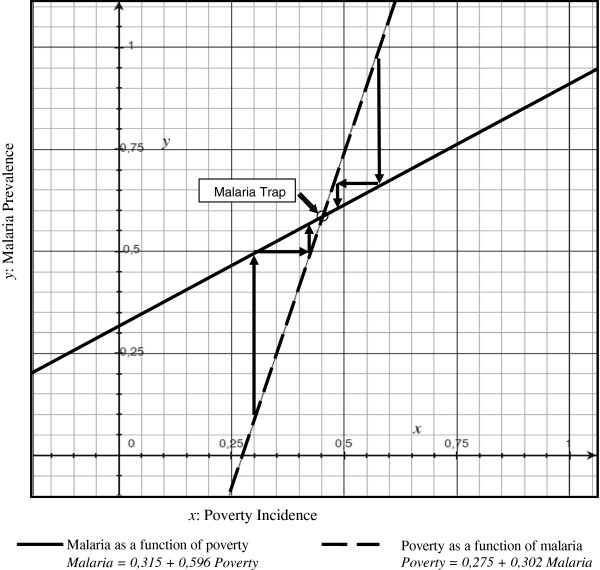
Malaria prevalence and poverty incidence in Uganda, solving partially the three-equations system.

By developing economic and econometric approaches on the basis of an epidemiological model, the poverty related-malaria trap has been formally determined. The combination of these three relations between protection, malaria and extreme poverty is, therefore, plausible and can lead to a malaria trap. Figure 2 illustrates the linear predictions of the relationship between malaria and poverty from Uganda dataset, solving partially the three-equations system (structural Equation (20)) to take into account the endogeneity of protection. It highlights three results with respect to possible traps.

First, for a poverty incidence equal to 0, malaria is persisting. Second, malaria can only converge to a medium or high equilibrium. Third, the intersection point between the x-axis and the poverty curve (as a function of malaria) is significantly higher (*p* < 0.001) than the intersection point between the x-axis and the malaria curve (as a function of poverty), which means that there is (at least) one stable equilibrium, with medium/high incidence of malaria and medium/high incidence of poverty. This stable equilibrium corresponds to the intersection of the two curves. Interestingly, the intersection between the two curves is slightly higher than the average level of malaria prevalence in Uganda (between 44% and 54% according to the DHS report [23]) and to the average incidence of poverty (28.67% according to the World Bank). It is, therefore, highly probable that communities/villages below this threshold will converge toward this point (i.e. a relatively higher prevalence of malaria/poverty equilibrium) and that communities/villages above this threshold will converge as well toward this point (i.e. a relatively lower prevalence of malaria/poverty equilibrium).

Social influences on individuals’ decisions may lead to malaria trap. This malaria trap can theoretically exist and the conditions of its existence have been identified, which provides policy implications. Particularly, the use of ITNs by the very poor should be subsidized, i.e. the very poor people should not only be provided highly subsidized ITNs, but they should be given incentive for protection use (including financial award) to keep and use their ITNs as suggested for immunization coverage in other empirical randomized studies [43]. Otherwise, they may rationally resell their ITNs on a parallel market (or use them for other purposes) and then malaria prevalence may stay high at equilibrium. It could be relevant to implement this policy at the community level in collaboration with community health workers, insofar as the origin of the issue is related to the presence of externalities that emerge at this community level. Obviously, ITNs or LLINs distribution should be complemented by insecticide spraying campaigns or other vector control methods, to reduce the number of vectors, and then the basic reproduction number. The model described in this paper could be extended in many ways. It would be interesting to distinguish asymptomatic infections from symptomatic infections in this model. Other potential external variables, such as drug and insecticide resistance, climatic variability, population immunity, access to care and other malaria preventive methods, could also be taken into account in a more complex model. Nevertheless, the result highlighted by this simple model has practical implication for malaria control policies. Indeed, these policies are based on combined actions, such as rapid diagnosis and Artemisinin Combination Therapy, preventive treatments (Seasonal Malaria Chemoprevention -SMC- and Intermittent Preventive Therapy during Pregnancy -IPTp), ITN/LLINs and environment management. The malaria trap illustrated in this study has to be taken into account when building malaria control policies.

## Competing interests

The authors declare that they have no competing interests.

## Supplementary Material

Additional file 1 Imperfect efficiency of ITN. Click here for file
