# Supplementary material for: Malaria and protective behaviours: is there a malaria trap?
Source: Malar J. 2013 Jun 13;12:200. doi: 10.1186/1475-2875-12-200 (PMC3691867; doi:10.1186/1475-2875-12-200)
Supplement: Additional file 1 — Imperfect efficiency of ITN. [file 1475-2875-12-200-S1.pdf]

Appendix: Imperfect efficiency of ITN

To take account of the imperfect efficiency of ITNs and LLINs, two definitions of efficiency are required.

First, the direct efficiency is defined as the probability of not being infected when using an ITN/LLIN. This parameter is termed ϕ_1 where $\phi_1 \leq 1$ and for perfect efficiency $\phi_1 = 1$. The probability of being infected when using an ITN/LLIN is, therefore, $(1 - \phi_1)\pi_I$ and the result of utility maximization becomes:

$h = 1$ if and only if

$$(1 - (1 - \phi_1)\pi_I)u(S) + (1 - \phi_1)u(I) - (1 - \pi_I)u(S) - \pi_I u(S) \geq \kappa W(\omega) \qquad (21)$$

Hence

$$h = 1 \text{ if and only if } \pi_I \geq \frac{C(\omega)}{\phi_1} \qquad (22)$$

In addition, equation (6) becomes:

$$X = (1 - \phi_1 H)\pi_I \qquad (23)$$

The effect of ITN efficiency can then be described by calculating the effect of ϕ_1 on X , by solving the following system at steady state for a variation of ϕ_1:

$$\begin{cases} \pi_i = Q(X, m(H)) \\ X = (1 - \phi_1 H)\pi_i \\ H = 1 - F(C^{-1}(\phi_1 \pi_I)) \end{cases} \qquad (24)$$

And thus,

$$\begin{cases} d\pi_i = \frac{\partial Q}{\partial X} dX + m' \frac{\partial Q}{\partial m} dH \\ dX = (1 - \phi_1 H) d\pi_i - \phi_1 \pi_i dH - H \pi_i d\phi_1 \\ dH = -\frac{f}{c'} \phi_1 d\pi_i - \frac{f}{c'} \pi_i d\phi_1 \end{cases} \qquad (25)$$

Note that increasing ϕ_1 in the second and third equations (i.e. in dX and dH) leads to an increase in *H* and a decrease in *X* (knowing that $f>0$ and $c'<0$) for a given π_i, and increasing *H* and decreasing *X* in the first equation ($d\pi_i$) lead to an increase in π_i, which enforces the

first effect. Hence for all values of π_I, X decreases with ϕ_1. Consequently, the possibility of a malaria trap is reinforced.

Second, the efficiency concerns the beliefs with respect to ITN efficiency: the expected probability of not being infected when using an ITN. This parameter is termed ϕ_2 where $\phi_2 \leq \phi_1$ and for expectation of perfect efficiency $\phi_2 = \phi_1$. Then the expected probability of being infected when using an ITN is $(1 - \phi_2)\pi_I$ and the result of utility maximization becomes:

$h = 1$ if and only if:

$$(1 - (1 - \phi_2)\pi_I)u(S) + (1 - \phi_2)u(I) - (1 - \pi_I)u(S) - \pi_I u(S) \geq \kappa W(\omega) \qquad (26)$$

Therefore

$$h = 1 \text{ if and only if } \pi_I \geq \frac{C(\omega)}{\phi_2} \qquad (27)$$

However, there is no change in equation (6), as steady state infection is determined by the probability of being infected at steady state:

$$\begin{cases} \pi_i = Q(X, m(H)) \\ X = (1 - H)\pi_i \\ H = 1 - F(C^{-1}(\phi_2 \pi_I)) \end{cases} \qquad (28)$$

The effect of beliefs about ITN efficiency can then be described by calculating the effect of ϕ_2 on X for a given level π_I, thus solving the following system:

$$\begin{cases} d\pi_i = \frac{\partial Q}{\partial X} dX + m' \frac{\partial Q}{\partial m} dH \\ dX = (1 - H)d\pi_i - \pi_i dH - H\pi_i \\ dH = -\frac{f}{c'}\phi_2 d\pi_i - \frac{f}{c'}\pi_i d\phi_2 \end{cases} \qquad (29)$$

Note that increasing ϕ_2 in the third equations (i.e. in dH) leads to an increase in *H* and thus a decrease in X in the second equation for a given π_i. As before, increasing *H* and decreasing *X* in the first equation ($d\pi_i$) lead to an increase in π_i, which enforces the first effect. Hence for all values of π_I, X decreases with ϕ_2. Consequently, the possibility of a malaria trap is reinforced.
